# Nanobiotechnology for the Therapeutic Targeting of Cancer Cells in Blood

**DOI:** 10.1007/s12195-015-0381-z

**Published:** 2015-02-24

**Authors:** Jiahe Li, Charles C. Sharkey, Dantong Huang, Michael R. King

**Affiliations:** Department of Biomedical Engineering, Cornell University, 205 Weill Hall, Ithaca, NY 14853 USA

**Keywords:** Circulating tumor cells, Metastasis, Nanomedicine

## Abstract

During metastasis, circulating tumor cells migrate away from a primary tumor *via* the blood circulation to form secondary tumors in distant organs. Mounting evidence from clinical observations indicates that the number of circulating tumor cells (CTCs) in the blood correlates with the progression of solid tumors before and during chemotherapy. Beyond the well-established role of CTCs as a fluid biopsy, however, the field of targeting CTCs for the prevention or reduction of metastases has just emerged. Conventional cancer therapeutics have a relatively short circulation time in the blood which may render the killing of CTCs inefficient due to reduced exposure of CTCs to drugs. Nevertheless, over the past few decades, the development of nanoparticles and nanoformulations to improve the half-life and release profile of drugs in circulation has rejuvenated certain traditional medicines in the emerging field of CTC neutralization. This review focuses on how the principles of nanomedicine may be applied to target CTCs. Moreover, inspired by the interactions between CTCs and host cells in the blood circulation, novel biomimetic approaches for targeted drug delivery are presented.

## Introduction

Metastasis contributes to more than 90% of cancer-associated mortality.^92^ It is generally hypothesized that primary tumors shed circulating tumor cells (CTCs) *via* the lymphatics to neighboring lymph nodes or through hematogenous dissemination to distant organs. The presence of CTCs in the blood represents a poor prognosis in a variety of carcinomas.^21^,^84^,^140^ Nevertheless, the effective treatment for this deadly disease remains clinically challenging. In the case of hematogenous metastasis, CTCs must complete several sequential steps: (1) detachment from the primary tumor, (2) intravasation into the vascular system, (3) survival in the blood circulation, and (4) extravasation into the target tissue.^41^ The finding that very few metastases develop despite the release of millions of CTCs into the vasculature daily by large primary tumors suggests that the process of metastasis is very inefficient.^76^ This is consistent with a recent experimental demonstration that only a small subpopulation of metastasis-initiating cells (MICs) among human luminal breast cancer CTCs gave rise to distant metastases in mice and the existence of MICs correlated with overall metastatic incidence in patients.^11^ Therefore, early metastasis intervention procedures, such as neutralization of CTCs and particularly MICs in circulation, may offer new therapeutic opportunities.

**Figure 1:**
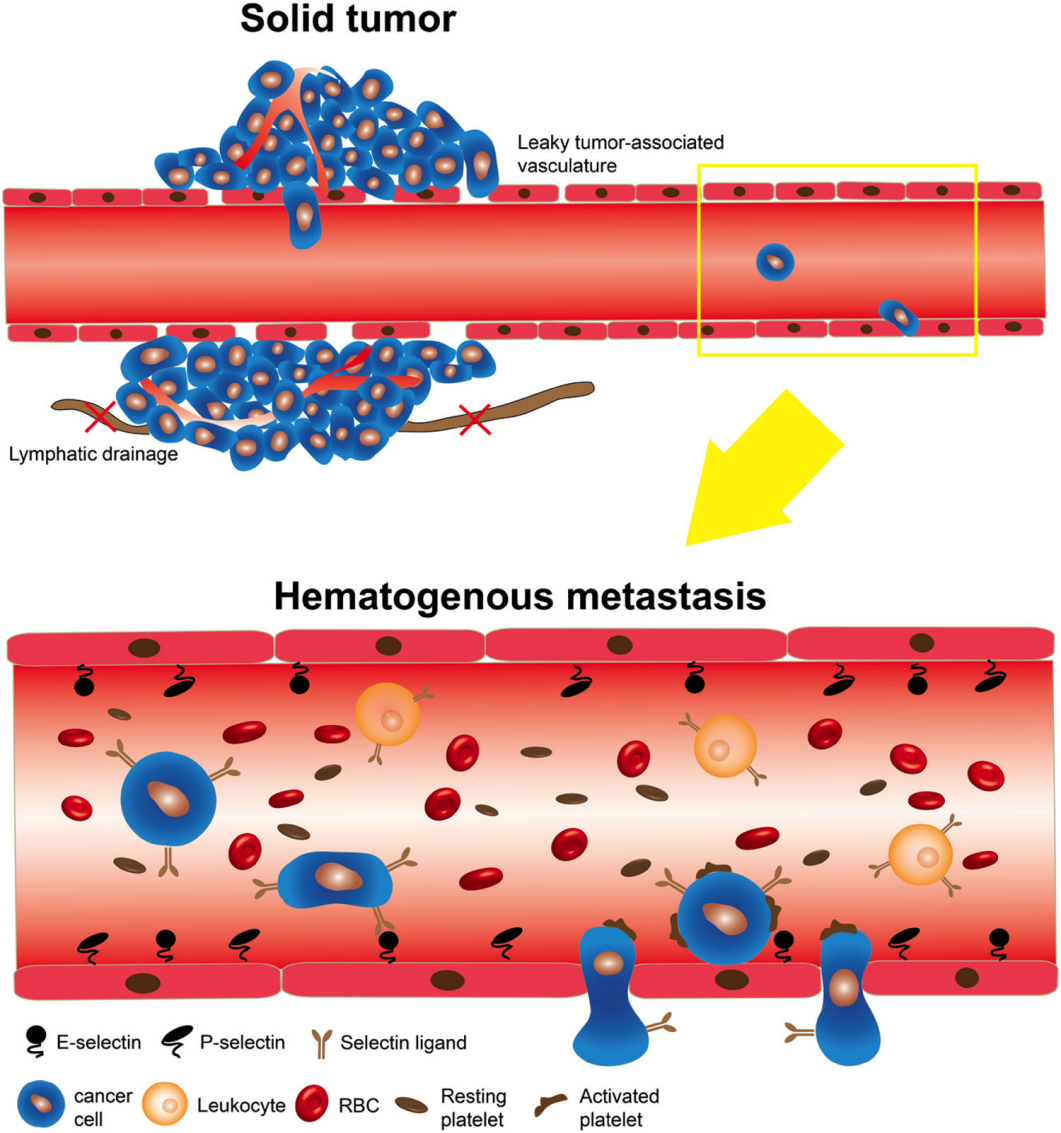
Two different contexts for the delivery of nanomedicines: solid tumor vs. the blood circulation. In a solid tumor, nanomedicine migrates to the tumor through leaky tumor-associated vasculature where dysfunctional lymphatic drainage enables accumulation of nanomedicine in the tumor. In contrast, once cancer cells are shed into the blood circulation to form CTCs, they are subjected to environmental changes such as shear stress, abundant RBCs and plasma proteins, and interactions with platelets, endothelial cells and other vascular components

The majority of existing cancer therapies including nanomedicine- and nanoformulation-based therapeutics target solid tumors (primary and metastatic).^23^ The underlying principle of most current nanotechnology-based drug delivery platforms is based on the observation that tumor-associated vasculatures are more leaky than normal vessels and thus are more permeable to nanoparticles and macromolecules. Additionally, solid tumors retain large molecules due to inefficient lymphatic drainage.^16^,^27^ While the enhanced permeability and retention (EPR) effect has proven to be a key pharmacokinetic feature for existing nanomedicines, this mechanism is not applicable to potential nanomedicines that target CTCs in circulation. The physical and biological environments surrounding CTCs are drastically different from those of solid tumors. CTCs are exposed to a broad range of fluid shear stresses when transiting in different vascular compartments (arteries, veins, and capillaries). Compared to non-transformed epithelial cells, transformed cells are remarkably resistant to varied fluid shear stress (FSS).^13^ In contrast, cancer cells in solid tumors are subjected to high interstitial fluid pressure caused by the stiff extracellular matrix.^59^,^129^ Additionally, certain CTCs gain the advantage of metastasis *via* interactions with different blood cells (neutrophils, macrophages, platelets, *etc*.) whereas solid tumors become aggressive by benefiting from hypoxia or certain tumor-promoting cells such as tumor-associated macrophages.^40^,^62^,^84^,^147^ These differences suggest that existing nanomedicines dependent on the EPR effect for targeted drug delivery must be tailored to the specific requirements for the neutralization of CTCs in circulation (Fig. 1).

In this review, we summarize the interactions of CTCs with different host cells during their hematogenous transit and how blocking CTC–host cell interactions *via* drug molecules have reduced metastases in animal models of cancer. Since the majority of these drugs suffer from fast clearance from circulation, methods to improve their circulation time through existing nanotechnologies will be discussed. Particularly, novel biomimetic CTC-targeting nanotechnologies are highlighted. Finally, potential CTC neutralization strategies that bridge conventional nanomedicine with technologies that are being utilized for CTC isolation and enumeration are discussed.

## Biology of Circulating Tumor Cells

### Aberrant Biological Events in CTCs

Solid tumors, at either primary or metastatic locations, can be accessed surgically in sufficient quantity for diagnostic tests and studies involving genomic sequencing, gene expression microarrays, immunohistology and mass spectrometry. In contrast, it remains a challenge to study the biology of rare CTCs in blood with conventional techniques. Efficient CTC isolation together with single-cell RNA-sequencing, exosome sequencing and immunofluorescence staining have enabled the discovery of several intracellular biological events underlying CTC-mediated metastasis. These events include but are not limited to: upregulation of Wnt2 in pancreatic cancer-derived CTCs,^155^ p53 mutation within CTCs of prostate cancer,^88^ activation of TGF-β and BMP signaling in CTCs from melanoma,^89^ and EGFR mutation in lung cancer CTCs.^34^ More generally, in nearly all epithelial cancers, a subpopulation of CTCs exist that are characterized by epithelial-to-mesenchymal transition (EMT) markers.^10^,^12^,^85^,^152^,^156^ During EMT, representative epithelial markers such as EpCAM and E-cadherin are downregulated, keratin expression pattern is altered, and mesenchymal markers such as N-cadherin, vimentin, and Snail are upregulated in turn.^63^,^115^ Entry to the mesenchymal state confers specific properties to CTCs including invasiveness, resistance to anoikis, chemo-resistance, and cancer stemness.^2^,^36^,^90^,^107^ Regardless of changes in signaling pathways and genomic integrity identified in CTCs, it remains to be answered whether these alterations merely mirror tumor progression and evolution occurring at primary and secondary tumors where CTCs are being shed. It has been postulated that subpopulations of metastasis-initiating cells (MICs) and cancer stem cells (CSCs) contribute to metastases in distant organs.^3^,^11^ It is of clinical significance if targeting of the aberrant signaling pathways could reduce the frequency of MICs and CSCs and thereby lead to better prognosis.

### Selectin-Mediated Hematogenous Metastasis

Selectins (L-, E-, and P-selectin) are integral membrane glycoproteins. They share several structurally similar domains: an N-terminal C-type lectin domain, an epidermal growth factor (EGF)-like domain, a variable number of short consensus repeats (2, 6, and 9 for L-, E-, and P-selectin, respectively), a single-pass transmembrane domain, and a short intracellular C-terminal tail.^83^ L-selectin is constitutively expressed on the surface of leukocytes whereas E- and P-selectin are restricted to inflamed endothelial cells, and P-selectin on activated platelets.^44^ It has been well accepted that during inflammation, the presence of E- and P-selectin on the endothelium causes the rolling and migration of neutrophils and monocytes which express selectin ligands.^123^,^158^

Selectin ligands, however, are not limited to leukocytes and they have been identified on the surface of certain cancer cells. Several lines of evidence support that both E- and P-selectin facilitate the rolling and adhesion of CTCs in hematogenous metastasis. Köhler *et al*. provided the first *in vivo* finding that E- and P-selectin are essential for colorectal cancer metastasis. In their study, E- and P-selectin double knockout mice had a 84% reduction of lung metastatic nodules by number compared to wild-type mice after they were subcutaneously implanted with HT29 colon cancer cells.^68^ Lung endothelial cells, however, do not constitutively express E- and P-selectin unless they receive inflammatory signals. It is not clear how selectins mediated the adhesion of HT29 cells to the lung endothelium. In contrast to the lungs, endothelial cells in the bone marrow constitutively express E-selectin.^49^,^146^ Studies have shown that E-selectin ligands on both human and mouse prostate cancer (PCa) cells facilitate bone metastasis in an E-selectin dependent manner.^15^,^86^,^150^ Instead of utilizing E-selectin knockout mice, these two studies overexpressed α-1,3 fucosyltransferases (FTs) in E-selectin ligand (ESL)-negative human and mouse PCa cell lines based on the findings that ESL-positive PCa cells highly express FT3, 6 or FT7. Consequently, these engineered PCa cells produced increased incidence of bone metastasis in mice. The role of specific selectin ligands in mediating the hematogenous metastasis of CTCs has been reviewed extensively elsewhere.^41^,^69^,^84^ This review, however, focuses on how nanobiotechnology can be utilized to inhibit the interaction between cancer cells and the endothelium for the prevention of metastasis.

Given that selectins recognize sialylated fucosylated glycans on selectin ligands such as sLe^*x*^, sLe^*x*^ analogs have been explored as competitive inhibitors for the binding of CTC selectin ligands to E- and P-selectin. For instance, a sLe^*x*^ analog, GSC-150, was tested for its effect on hepatic metastasis of human colon carcinoma in nude mice. It was found that liver metastases were significantly attenuated when cancer cells were co-administered with GSC-150.^130^ In addition to sLe^*x*^ analogs, compounds that can interfere with the synthesis of sLe^*x*^ represent another class of selectin inhibitors. We previously showed that a fluorinated fucose mimetic (2F-Peracetyl-Fucose) could be used to reduce E-selectin-dependent bone metastasis in mice by inhibiting the activity of FT6.^86^

### Contribution of Platelets to CTC-Mediated Metastasis

The involvement of platelets in cancer was first reported in the mid-nineteenth century by the French clinician Armand Trousseau.^141^ He diagnosed patients with migratory thrombophlebitis caused by an occult visceral carcinoma. Mounting evidence has shown that the interaction of platelets with tumor cells can promote metastasis through several mechanisms. For instance, interactions with platelets protect CTCs from immune-mediated clearance,^77^,^102^,^106^ because such adhesion events affect the recognition of CTCs by natural killer cells. Platelet aggregation can result in the grafting of MHC class I ligands onto CTCs, which are typically absent. The newly acquired ligands prevent natural killer cells from identifying the CTCs as “non-self” and spare them from attack.^114^ Additionally, activated platelets can induce EMT as well as pro-survival and pro-metastatic signaling in tumor cells, which are associated with enhanced invasiveness and metastatic potential.^35^,^74^,^75^

It has been demonstrated that therapies targeting the interactions between platelets and CTCs can reduce the formation of secondary metastases. One approach to minimizing these interactions has focused on utilizing anti-coagulation agents. Agents such as recombinant mouse tissue factor pathway inhibitor (TFPI) and Cilostazol have been found to reduce the formation of secondary metastases.^8^,^9^,^145^ Unfortunately, the use of anticoagulants may also adversely affect the normal hemostatic function of platelets in the case of bleeding. A more focused approach aims to block the signaling between platelets and CTCs. Invasive behavior can be induced by transforming growth factor-β1 (TGF-β1), which is secreted by activated platelets. Temporary contact with platelets is sufficient to induce invasive behavior in CTCs through TGF-β1.^74^ Blockage of TGF-β1 receptor I (TβR1) kinase activity through the use of SD-208, a small molecule inhibitor, was shown to prevent the development of TGF-β induced bone metastases in a melanoma mouse model.^100^ Thus, blocking platelet-CTC signaling is a potentially viable targeted therapy to prevent the formation of additional metastases.

## Challenges of Targeting CTCs by Nanomedicine

Although nanomedicine is a very general concept and represents a broad range of nanoformulations for existing drugs, it manifests itself largely in targeted drug delivery and controlled release. The biological characteristics of CTCs and the physical environment where they reside may pose certain challenges and problems when targeted by nanomedicine (Table 1). Searching for a rare number of CTCs in circulation is likely analogous to the problem of finding a needle in a haystack.^5^,^47^ This challenge may be solved by adopting the strategies utilized for CTC capture and enrichment. For example, targeting moieties for CTC isolation, such as anti-EpCAM monoclonal antibody, can be functionalized onto nanoparticles for the recognition of CTCs with epithelial origin in the circulation.^28^

**Table 1:** Challenges of targeting CTCs by nanomedicine.

CTC properties	Practical consequences	Refs.
Rarity of CTCs: a needle in a haystack problem	Low efficiency of targeting CTCs	5,47
Heterogeneous subpopulations	Mesenchymal CTCs are not recognized by nanomedicine targeting epithelial cell markers such as EpCAM; Necessity of killing all CTCs vs. MICs or CSCs in the circulation; Differential drug resistance among different subpopulations	73,78,149,161
Formation of CTC clusters	Increased invasiveness, resistance to anoikis and trapping in microvessels	1,24,46,66,163
Short circulation time of CTCs	Limited exposure time to therapeutics against CTCs in circulation	93,125,135
Shielding of CTCs by platelets	Physical barrier to penetration of nanomedicine into CTCs; pro-metastatic role *via* induction of EMT, establishment of early metastatic niches, pro-survival signaling *etc*.	35,74,75
Off-target effects associated with systemic drug delivery	Systemic cytotoxicity	136,162

The heterogeneity of CTCs, however, dictates that there is no universal antigen for comprehensive targeting. CTC phenotypes can be categorized into epithelial (epithelial^+^/mesenchymal^−^), complete EMT (epithelial^−^/mesenchymal^+^), and intermediate EMT (epithelial^+^/mesenchymal^+^).^73^,^78^ It is possible that all three phenotypes exist in the circulation simultaneously and thus targeting CTCs with epithelial features may become ineffective against those with mesenchymal characteristics.^161^ Moreover, CTCs can also be divided into CSCs and non-CSCs according to their tumor-initiating capability, which is not necessarily coupled to the EMT status.^149^ This raises the question of whether it is necessary to neutralize all CTCs in circulation to achieve a net reduction in metastasis.

CTCs have been detected both as individual cells and as cellular clusters in blood.^24^,^66^ Although rare in circulation compared to individual CTCs, CTC clusters show increased invasiveness, are more resistant to anoikis and have a higher likelihood of becoming trapped in microvessels, thereby favoring their survival and extravasation into distant organs.^46^,^163^ In a study using breast cancer CTCs, it was been found that CTC clusters display a 23- to 50-fold increase in metastatic potential. Moreover, high expression of the cell junction protein plakoglobin was identified as responsible for this intercellular adhesion.^1^ In addition to promoting invasiveness, it is reasonable that, in large cell aggregates, the cells within the core may be less accessible to nanomedicine approaches compared to those at the periphery.

Recently, *ex vivo* culture of breast cancer CTCs has enabled individualized testing of drug susceptibility.^157^ Although such a strategy is mainly utilized for the treatment of solid tumors, which are the origin of the CTCs expanded *ex vivo*, it remains unclear whether the drugs selected from such screening would be effective against CTCs in blood compared to the CTC cell lines expanded in culture medium. In light of several protective effects associated with the adhesion of platelets to CTCs in blood as discussed in the “Contribution of Platelets to CTC-Mediated Metastasis” section, platelet shielding may not only provide a physical barrier to nanomedicine-mediated drug delivery to CTCs, but also potentially confer drug resistance.

The systemic dissemination of CTCs defines a requirement that nanomedicine vehicles must exist in the circulation for an extended period of time to patrol for metastatic CTCs released from solid tumors. When conventional cancer drugs with systemic cytotoxicity such as doxorubicin and paclitaxel are utilized for CTC neutralization, they should ideally be released only after nanocarriers encapsulating the drugs have been internalized into CTCs to avoid off-target effects on normal tissues.^136^,^162^ In contrast, when more cancer-specific therapeutics such as monoclonal antibodies and tumor necrosis factor-related apoptosis-inducing ligand (TRAIL) are applied to target CTCs, such systemic toxicity may be less of a concern.^70^,^126^,^143^ In addition to their systemic nature, CTCs are reported to have a relatively short half-life (<24 h) in circulation.^93^,^125^,^135^ This provides a narrow time window for efficient killing of CTCs *via* nanomedicine approaches. A rational solution to this challenge is to design nanocarriers with extended circulation times. Recently, our group demonstrated for the first time that leukocytes coated with nanoscale TRAIL-liposomes were able to efficiently neutralize CTCs in an experimental mouse model of metastasis. This approach utilizes the long circulation time of leukocytes to deliver an apoptosis signal to cancer cells with minimal side effects on normal cells such as leukocytes and endothelium.^95^ An alternative strategy, however, is to deliver drugs that inhibit intravasation of cancer cells *via* enhanced tumor cell–matrix interactions.^164^

## Potential Paradigms of Conventional Nanomedicine in CTC Neutralization

Despite the promise of utilizing synthetic compounds for blocking selectin-mediated CTC metastasis or inhibitors for blocking platelet–CTC interactions and signaling, these compounds are likely to be cleared from the body within a short time period *via* renal filtration due to their relatively low molecular weight (LMW).^87^ Therefore, frequent administration of these molecules is necessary and may become prohibitively expensive for clinical implementation. Nevertheless, the paradigms that have been exploited for nanoparticles and nanoformulations over the past few decades are potentially applicable to extend the circulation time of these LMW compounds. To date, two nanoformulations for treating solid tumors have been approved for clinical use, specifically liposomal doxorubicin (Doxil) and protein-bound paclitaxel (Abraxane).^37^ One major obstacle to using nanoparticles *in vivo* is rapid clearance by the mononuclear phagocyte system (MPS). The main strategy for extending the circulation time of nanoparticles is by grafting uncharged hydrophilic polymers onto the surface of particles to create so-called “stealth” particles, with the most commonly used polymer being polyethylene glycol (PEG).^110^,^116^,^127^ By encapsulating sLe^*x*^ analogs or fucosyltransferase inhibitors into nanoparticles with PEG-coated surfaces, this is likely to achieve both controlled drug release and extended circulation time. In addition to the intervention of selectin-mediated adhesion of CTCs to endothelium *via* ESL inhibitor-encapsulated nanoparticles, a coexisting or alternative adhesion event mediated by ICAM-1 (expressed on vascular endothelium) and MUC1 (expressed on some CTCs) has been shown as a potential target for the prevention of metastasis.^117^,^121^ The Decuzzi lab recently demonstrated that long circulating lipid–polymer nanoparticles encapsulating curcumin were able to substantially reduce the adhesion of highly metastatic human breast cancer cells MDA-MB-231 to TNF-α-treated HUVEC cells in an ICAM-1- and MUC1-dependent manner.^111^ It remains to be determined, however, whether such curcumin-encapsulated nanoparticles would be effective in mouse models with experimental and/or spontaneous metastases.

Alternatively, sLe^*x*^ or synthetic sLe^*x*^ analogs can be functionalized onto the surface of nanoparticles for targeting tumor-associated endothelium. For instance, sLe^*x*^-conjugated liposomes loaded with cisplatin have been shown to accumulate on an E-selectin-expressing endothelium in the vicinity of tumor cells. It was found that sLe^*x*^-conjugated liposomes enabled sixfold higher cisplatin accumulation than non-conjugated liposomes in tumors.^45^ Although this nanoformulation was intended to target solid tumors as opposed to CTCs, an additional benefit may have been to help neutralize CTCs that reseed into solid tumors.^65^ Bone-metastatic prostate cancer (PCa) cells bearing ESLs are known to attach more avidly to bone marrow (BM) endothelial cells which constitutively express E-selectin.^14^,^86^ Therefore, sLe^*x*^-conjugated nanoparticles encapsulating cancer therapeutics can potentially deliver conventional cancer drugs to the BM niche of CTCs for the prevention and treatment of BM metastasis in prostate cancer.

## Advanced Nanotechnologies in CTC Targeting

### Effect of Nanoparticle Morphology on Their Fate in Blood Circulation

Many nanoparticle designs have been proposed for cancer therapy and diagnosis, with varied biodistribution in the blood circulation depending on their administration route, particle size, composition, and surface charge.^18^ In general, polymeric nanoparticles less than 10 nm in diameter may be easily cleared by the kidneys as blood carries them through the renal system, and particles greater than 100 nm may be easily cleared by phagocytic uptake and hepatic filtration.^4^ For the targeting of CTCs that extravasate from the vessel wall of the microvasculature, nanoparticles that exhibit the capability to adhere to the vascular wall may be more effective at delivering therapeutic cargo to CTCs.^122^ Gentile *et al.* 43 have shown that for spherical particles, large particles will have better margination compared to small particles that are less than 200 nm in diameter. Margination is a biorheological process in which semi-rigid cells and particles are displaced toward the vessel wall in circulation, providing more opportunities for interactions with the endothelium^43^ such as CTC intravasation and extravasation and blockade of these processes. The shape of nanoparticles is an important factor in determining their behavior in the blood circulation, where the adhesive interactions between particles and cells have to counteract the hemodynamic forces exerted by the flowing blood.^29^ These two counteracting forces will affect the particles’ targeting and attachment abilities within the microvasculature. Various shapes of nanoparticles, such as spherical, hemispherical, discoidal, cylindrical, conical, vase- and rod-shaped, have all been manufactured with emerging nanofabrication technologies and have demonstrated differential behaviors in flow.^22^,^30^

In flowing blood, a spherical morphology is suited for rotational motion,^58^ which is important for leukocytes that roll on and interact with the endothelium. In contrast to symmetric spherical particles (including cells), non-spherical particles may align or tumble under flow, with surprising transport properties. For example, red blood cells (RBCs), having a flexible biconcave disc shape with an average diameter of 8 *μ*m,^108^ routinely pass through the reticular meshwork filtering units in the sinusoidal spleen in which the cell slit size rarely exceeds 200–500 nm in width, whereas spherical nanoparticles must be less than 200 nm in diameter to do so.^22^,^99^ In contrast to rigid spheres, biconcave discs are found to deform to other shapes such as parachute and slipper-like morphologies in response to changes in local flow velocity and shear stress, yet they retain the ability to recover their discoidal shape at reduced velocity.^108^ Shape flexibility and deformability allow RBCs to pass through vessels of different dimensions and narrow constrictions, making them an excellent vehicle for traveling in blood. Another important type of blood cell, platelets, normally exhibiting an oblate spheroidal shape, have been shown to assume different morphologies *in vitro* after prolonged exposure to adhesive surfaces.^58^ Activated platelets resemble spheres with a rough surface. The activated platelet shape greatly influences platelet collisions, including the frequency, contact time and available area of collision, as well as the magnitude of shear and normal forces acting on the cells.^98^

Inspired by nature’s adaptation of non-spherical particles for unique transport properties and cellular interactions in flowing blood, synthetic particles of various shapes have been developed and evaluated for drug delivery in recent years.^29^ For instance, Decuzzi *et al.* 30 injected silicon-based particles of quasi-hemispherical, cylindrical, and discoidal shapes into tumor-bearing mice and observed different distribution profiles among these particles. They further showed that discoidal particles can maximize accumulation in target organs while reducing sequestration by the liver. Geng *et al.* 42 prepared filomicelles, which are flexible filamentous vehicles that have been shown to effectively and efficiently deliver the anticancer drug paclitaxel to tumors in mice,^20^ that persist in circulation 10 times longer than their spherical counterpart. Gandra *et al.* 38 modified a filamentous bacteriophage and demonstrated its potential use as a biological nanowire to convey cargos of cancer-targeting peptides and photosensitizing agents. Bruckman *et al.* 19 prepared viral nanoparticles in the forms of rods and spheres, and observed that the nanorods circulated longer in the bloodstream of mice and were cleared from tissues more slowly compared to nanospheres. Theoretical and experimental calculations further confirmed the distinctive diffusion profiles of these two shapes in the tumor microenvironment.^82^ Carbon nanotubes (CNTs) have attracted much attention in drug delivery research due to their long circulation time and the efficient methodologies for chemical modification.^17^ For example, Yinghuai *et al.* 151 constructed water-soluble functionalized CNTs as delivery vehicles of cancer therapeutics—BNCT agents—which were found to be concentrated in tumor cells in mice.

These bio-inspired approaches exploiting non-spherical particles for tumor homing are directly applicable for the targeting of CTCs in the bloodstream. Elongated, rod-shaped and filamentous materials possess distinctive transport properties compared to spheres due to their enhanced flexibility and permeability. They also show improved margination toward the vessel wall,^79^,^82^ and are thus potentially more effective at accessing diseased vessels and CTCs.^82^ Moreover, the extended circulation lifetime of these particles can increase their probability of interaction with CTCs. Although non-spherical nanoformulations are promising anticancer drug delivery vehicles, they represent an emerging field that requires more extensive evaluations, including focus on mechanical properties, polydispersity, and stability of the carriers. In addition, other design parameters can have significant effects on vascular transport properties as well. For example, size and density are important in particle design. A fine balance among these three factors—shape, size and density—may allow for the design of particles with enhanced vascular interactions^80^ that are able to mimic biomolecules and cells, sensing and interacting with endothelial cells and CTCs in the blood circulation.

### Biomimetic Strategies for Drug Delivery to CTCs

Red blood cells (RBCs), or erythrocytes, have been exploited as drug delivery vehicles since Inler *et al*.^54^ first created enzyme-loaded RBC ghosts in the early 1970s.^104^ Their work continues to inspire the design and engineering of biomimetic delivery systems today. Drug delivery vehicles derived from natural RBCs can be divided into four major classes:^50^ (1) carrier RBCs, which are natural RBC ghosts carrying therapeutic cargos; (2) synthetic RBC-mimicking particles, which are made of polymers that aim to simulate the mechanical and chemical properties of RBCs; (3) RBC membrane-derived liposomes, which are synthesized from native RBC membranes; and (4) RBC-membrane camouflaged nanoparticles (RBC-NPs), which are nanoparticles coated with native RBC membranes.

Although RBC derivatives have not been extensively evaluated in the context of CTCs, their unique biomimetic features suggest they are an excellent type of delivery vehicle for drugs that are intended to act in the bloodstream,^104^ and exhibit the potential to effectively target CTCs. Both RBCs and CTCs reside in the circulatory system, and can travel to different organs through blood vessels. Therefore, if bioengineered RBCs are capable of recognizing and eliminating CTCs in the vasculature before CTCs are able to extravasate, they could prevent cancer cells from colonizing secondary organs and reduce metastasis. In addition, human erythrocytes have a life span of 100–120 days,^104^ a circulation time much longer than that of nanoparticle drug carriers at present. Many types of synthetic nanoparticles sub-100 nm in diameter have a circulation half-life on the scale of hours, even after PEGylation.^60^ The longer blood circulation time of RBC derivatives enhances drug retention in the body and allows for sustained drug release,^51^ as well as increasing the vehicles’ interactions with CTCs.

Another property that makes RBC derivatives an excellent tool for targeting CTCs is their superior biocompatibility. The biocompatibility of nanoparticles is dictated by particle size, surface charge, hydrophobicity-hydrophilicity, as well as the steric effects of their outer coating.^166^ Many polymers used for nanoparticle stealth coating, such as PEG, create a hydrophilic shell on the particle surface, thus shielding the nanoparticles from immune recognition and decreasing their rate of elimination.^55^ In the early 2000s, however, different groups reported a phenomenon called “accelerated blood clearance”,^26^ in which repeated injections of PEGylated liposomes resulted in immune rejection in animal studies.^26^,^55^–^57^ This phenomenon raised concerns in the repeated administration of sterically stabilized nanoparticles for drug delivery. In contrast to PEGylation, which provides nanoparticles with an outer shell that attenuates immune recognition, RBC derivatives adopt a different mechanism by disguising as the “self”, which can potentially be more compatible with the immune system and avoid accelerated blood clearance. Residing in the same environment as macrophages and lymphocytes, RBCs evade the immune system by displaying self-antigens on their outer membrane.^50^,^64^,^109^ RBC derivatives that incorporate self-antigens, such as CD47,^48^ or a complete RBC membrane,^39^,^50^,^51^ have shown reduced immunogenicity compared to naked particles. For instance, Gao *et al.* 106 demonstrated a fourfold reduction in the uptake of gold nanoparticles (AuNP) coated with RBC membrane by macrophages *in vitro*.

Early studies have shown enhanced therapeutic efficacy and reduced immunogenicity in animal cancer models treated with erythrocyte-encapsulated antitumor drugs. In a study by Zocchi *et al.*,^165^ murine RBCs were subjected to hypotonic dialysis followed by doxorubicin encapsulation during membrane resealing. Compared to the non-encapsulated drug, mice treated with erythrocyte-encapsulated doxorubicin showed significant inhibition of metastatic growth in liver and lung at a much lower dosage. Doxorubicin encapsulated in RBCs was also administered to dogs with lymphosarcoma.^91^ This treatment achieved sustained drug release and induced complete and partial remissions of lymphosarcoma in dogs. Skorokhod *et al.* 133 administered doxorubicin-loaded erythrocytes to 15 lymphoma patients and reported improved pharmacokinetics compared to those of free doxorubicin, as well as good tolerance in cancer patients. The same research group has also studied the pharmacokinetics of daunorubicin-loaded erythrocytes in patients with acute leukemia^132^ and similar findings were reported, thus demonstrating the promising clinical applications of RBC delivery vehicles in treating blood cancers. Another drug used to treat acute leukemia, L-asparaginase, is one of the most widely studied enzymes for RBC encapsulation.^94^ Different groups have developed various methods for preparing asparaginase-loaded RBCs, which have been evaluated for their pharmacokinetics and antitumor activities in mice,^7^,^71^ dogs,^31^ monkeys^139^ and humans.^72^ These studies have demonstrated the advantages of using RBC derivatives for targeting CTCs *in vivo*.

To date, RBC derivatives have not been reported in the literature for targeting CTCs that are not of blood cancer origin. Nevertheless, studies involving leukemia or lymphoma animal models and human patients have corroborated the potential use of RBC carriers in targeting non-blood CTCs. Despite the attractive features of erythrocyte derivatives in targeted drug delivery, there are still many clinical challenges. Unlike animals, humans have many different blood groups. To make the technology more versatile for patients, the removal of immunogenic antigens is essential during drug synthesis.^51^ In addition, because of their biological origin, RBC derivatives are difficult and expensive to store. They also present great variability, which makes standardization and scale-up challenging.^94^ At present, there is no known receptor or ligand on the native RBC membrane that would allow RBC vehicles to interact with CTCs in the circulation. A potential solution is to create RBC derivatives carrying antibodies that would specifically recognize CTC surface antigens such as EpCAM, which is present in many types of CTCs. EpCAM expression on CTCs is also found to correlate with metastatic cancer prognosis.^120^,^124^ Conjugation of various molecules, including antibodies, to RBC membranes can be accomplished through a biotin-avidin linkage.^50^,^103^

In contrast to RBC biomimetic platforms that primarily seek to improve drug circulation time and biocompatibility, nanoparticles that mimic behaviors of platelets may not only extend the half-life of particles in blood but also bind CTCs with mechanisms similar to natural platelets. A platelet-mimetic approach for metastasis-targeted nanomedicine has been recently developed. In a study from the Gupta group,^97^ highly metastatic human breast cancer cells MDA-MB-231 were examined for surface expression of platelet-interactive receptors, which were then compared to a weakly metastatic human breast cancer cell line, MCF-7. Interestingly, certain platelet-interactive receptors were found to be significantly overexpressed on the surface of MDA-MB-231 cells such as GPIIb-IIIa-like receptors (which can bind to platelets mediated by fibrinogen), P-selectin (which can bind to platelets mediated by sialoprotein ligands), GPIa-IIa-like receptors (which can bind to platelets mediated by collagen-like molecules), E-selectin (which can bind to sialyl Lewis moieties), integrin α_V_β_3_ (which can bind to fibronectin, vitronectin, *etc*.), and GPIbα-like proteins (which can bind to von Willebrand factor). In contrast, these receptors were weakly expressed in MCF-7 cells. More importantly, MDA-MB-231 cells showed significantly enhanced binding interactions with active platelets compared to MCF-7 cells. In light of these differences, two specific receptors were selected (GPIIb-IIIa-like integrin and P-selectin), and their corresponding ligands were engineered onto the surface of liposomes to enable platelet-mimetic binding to the cancer cells under physiological flow conditions. Nevertheless, it remains to be answered whether such a platelet-mimetic approach could target real CTCs in a patient’s blood. To address this question, both a mouse metastatic cancer model and patient-derived blood containing CTCs will need to be tested. In addition, the *in vivo* biodistribution and circulation time of such nanomedicines must be measured. Despite the uncertainties that remain to be addressed, this represents the first paradigm for targeting metastatic CTCs though interactions with platelets.

## Integrating CTC Isolation Technologies with Nanomedicine

Enumeration of CTCs in the peripheral blood of cancer patients has shown promise for the diagnosis and monitoring of cancer progression, and it serves as an alternative to conventional imaging methods.^61^,^96^,^154^ CTCs of epithelial origin are defined as being positive for epithelial cell adhesion molecule (EpCAM^+^) and cytokeratin 8, 18, or 19 (CK^+^), and negative for CD45 (CD45^−^).^6^ Therefore, approaches for isolating such CTCs are largely based on the positive selection for epithelial markers.^25^,^138^ The existence of EpCAM-negative CTCs (i.e., CTCs with EMT signature), necessitates approaches such as negative depletion to select for CD45^−^ cells.^33^ Alternatively, label-independent enrichment methods that are based on size and/or density differences between cancer cells and blood cells have also been developed for CTC enrichment.^112^,^142^ In addition to cell heterogeneity, the rarity of CTCs in blood relative to white blood cells has made their detection and isolation even more challenging.^5^,^10^,^47^ To solve this problem, nanomaterials have been developed to enable high-density coatings of different capture molecules such as antibodies for improved sensitivity of CTC detection.^148^ Recently, silicon nanopillars,^144^ quartz nanowires^81^ and TiO_2_ nanofibers^159^ have been used to trap CTCs, with enhanced capture efficiency due to the higher aspect ratio of the nanomaterials. Moreover, when such nanomaterial-based platforms have been integrated with flow-based systems such as microfluidic devices, a significant increase in capture yield is observed *via* continuous flow of patient blood through the devices.^32^,^105^,^153^

In contrast to the rapid development of CTC isolation technologies, few studies have been performed to enable drug delivery through CTC enrichment devices. It is conceivable that anti-EpCAM-conjugated nanoparticles loaded with cancer drugs could be utilized for targeting CTCs of epithelial state in blood. Moreover, by combining high capture efficiency of 3D nanotopographic features such as silicon nanowires with enhanced drug encapsulation for nanoporous materials, novel nanomedicines can possibly target a subpopulation of CTCs with CSC properties or multi-drug resistance.^67^,^113^,^128^ In addition, since mouse models and clinical observations have provided evidence that CTC count correlates with disease progression in cancer patients,^28^,^65^,^154^,^160^ it is intriguing that an implanted shunt system with similar nanostructure and surface functionalization could be utilized to filter out rare CTCs from the blood circulation. As a proof-of-concept, our group invented a biomimetic approach to capture and kill CTCs *in vitro*. In this system, a microfluidic device was functionalized with E-selectin, which interacts with CTCs during extravasation through the endothelium. Additionally, the surface was coated with a tumor-specific cytokine, tumor necrosis factor-related apoptosis-inducing ligand (TRAIL), for inducing apoptosis when CTCs were captured from the flow.^118^,^119^ More recently, the device was further functionalized with naturally occurring nontoxic halloysite nanotubes for the enhanced capture of CTCs.^52^,^53^ Nevertheless, several issues need to be addressed to demonstrate the efficacy of this approach *in vivo*. First, the device must be compatible with the body. As E-selectin and TRAIL are expressed in host cells, it is unlikely that they will induce an immune response. It requires, however, further examination on whether the material that comprises the device and additional nanostructured surface would cause any side effects when interfacing with blood.^131^ Secondly, it is necessary to test the isolation efficiency of CTCs in the presence of abundant plasma proteins in the blood. It has been found that owing to high surface free energy, certain nanomaterials adsorb biomolecules upon contact with biological fluids. In particular, plasma proteins may bind to the nanostructured surface to form a biological coating, known as the protein corona.^101^,^137^ This corona may affect the interaction of the device with the host system.^134^ Lastly, it is crucial to evaluate when the device surface becomes saturated with CTCs and requires replacement. Nevertheless, such biomimetic approaches for the delivery of apoptotic signals represents an intriguing proof of concept for future integration of CTC isolation technologies with nanomedicines.

## Conclusion

In the past decade, the presence of CTCs has been utilized as an indicator of poor prognosis in several carcinomas. The difficulty of identifying these rare cells in the blood has driven the development of numerous devices for the isolation and characterization of CTCs in clinical settings. It is not until recently, however, that the presence of metastasis-initiating cells (MICs) among CTCs has been experimentally demonstrated. Therefore, neutralizing MICs or CTCs in the blood may represent a new paradigm for the intervention of metastases in distant organs. In contrast to conventional nanomedicines, which extend the half life of chemotherapeutics in the blood, novel approaches that are inspired by the context of CTCs in circulation have led to a variety of biomimetic nanoparticle or nanoformulation platforms. Integration of nanotechnologies with a deeper understanding of diverse CTC–host cell interactions may offer exciting and promising directions for novel therapeutic interventions in the future.
